# Prioritizing Parkinson’s disease risk genes in genome-wide association loci

**DOI:** 10.1101/2024.12.13.24318996

**Published:** 2024-12-14

**Authors:** Lara M. Lange, Catalina Cerquera-Cleves, Marijn Schipper, Georgia Panagiotaropoulou, Alice Braun, Julia Kraft, Swapnil Awasthi, Nathaniel Bell, Danielle Posthuma, Stephan Ripke, Cornelis Blauwendraat, Karl Heilbron

**Affiliations:** 1 Institute of Neurogenetics, University of Luebeck, Luebeck, Germany; 2 Laboratory of Neurogenetics, National Institute on Aging, Bethesda, Maryland, USA; 3 Neurology Unit, Department of Neurosciences, Hospital Universitario San Ignacio, Bogotá, Colombia.; 4 CHU de Québec Research Center, Axe Neurosciences, Laval University, Quebec City, Quebec, Canada.; 5 Vrije Universiteit Amsterdam, Amsterdam, The Netherlands; 6 Department of Psychiatry and Psychotherapy, Charité – Universitätsmedizin Berlin, Berlin, Germany; 7 Stanley Center for Psychiatric Research, Broad Institute of MIT and Harvard, Cambridge, Massachusetts, USA; 8 German Center for Mental Health (DZPG), partner site Berlin/Potsdam, Berlin, Germany; 9 Department of Child and Adolescent Psychiatry and Pediatric Psychology, Section Complex Trait Genetics, Amsterdam Neuroscience, Vrije Universiteit Medical Center, Amsterdam, The Netherlands; 10 Center for Alzheimer’s and Related Dementias, National Institute on Aging and National Institute of Neurological Disorders and Stroke, National Institutes of Health, Bethesda, MD, USA; 11 Current address: Bayer AG, Research & Development, Pharmaceuticals, Berlin, Germany

**Keywords:** Parkinson’s disease, genome-wide association study, gene prioritization, statistical genetics, PoPS

## Abstract

Recent advancements in Parkinson’s disease (PD) drug development have been significantly driven by genetic research. Importantly, drugs supported by genetic evidence are more likely to be approved. While genome-wide association studies (GWAS) are a powerful tool to nominate genomic regions associated with certain traits or diseases, pinpointing the causal biologically relevant gene is often challenging. Our aim was to prioritize genes underlying PD GWAS signals.

The polygenic priority score (PoPS) is a similarity-based gene prioritization method that integrates genome-wide information from MAGMA gene-level association tests and more than 57,000 gene-level features, including gene expression, biological pathways, and protein-protein interactions. We applied PoPS to data from the largest published PD GWAS in East Asian- and European-ancestries.

We identified 120 independent associations with *P* < 5×10^−8^ and prioritized 46 PD genes across these loci based on their PoPS scores, distance to the GWAS signal, and presence of non-synonymous variants in the credible set. Alongside well-established PD genes (*e.g., TMEM175* and *VPS13C*), some of which are targeted in ongoing clinical trials (*i.e*., *SNCA*, *LRRK2*, and *GBA1*), we prioritized genes with a plausible mechanistic link to PD pathogenesis (*e.g., RIT2, BAG3*, and *SCARB2*). Many of these genes hold potential for drug repurposing or novel therapeutic developments for PD (*i.e., FYN, DYRK1A, NOD2, CTSB, SV2C*, and *ITPKB*). Additionally, we prioritized potentially druggable genes that are relatively unexplored in PD (*XPO1, PIK3CA, EP300, MAP4K4, CAMK2D, NCOR1*, and *WDR43*).

We prioritized a high-confidence list of genes with strong links to PD pathogenesis that may represent our next-best candidates for disease-modifying therapeutics. We hope our findings stimulate further investigations and preclinical work to facilitate PD drug development programs.

## Introduction

Parkinson’s disease (PD) is a neurodegenerative condition with the fastest-growing global prevalence among all neurological disorders, posing significant challenges to healthcare systems and society^1^. The pathophysiology of PD involves multiple risk factors, including genetics, environmental factors, and aging^2^. Currently, most therapeutic strategies aim to address dopamine deficiency (*e.g*., levodopa or dopamine agonists). More advanced therapies, such as deep brain stimulation, focus on modulating neural synchrony in distributed brain networks^3^. Although these treatments are intended to alleviate symptoms, they do not clearly impact disease progression^4^. Hence, there is an urgent need to identify specific therapeutic targets that can effectively halt the course of the disease and accelerate the clinical development of disease-modifying therapies.

Recent advancements in drug development for PD have been significantly influenced by over two decades of genetic research. This body of evidence has not only deepened our understanding of the biology underlying PD, but has also led to the identification of numerous drug targets. Some of these are currently being evaluated in clinical trials^5^, including *GBA1*, *LRRK2* and *SNCA*. Drugs supported by genetic evidence are more than twice as likely to be approved^6,7^, and human genetic data supported two-thirds of the drugs approved by the FDA from 2013 to 2022^8,9^. Leveraging data from genetic studies, and developing pipelines to prioritize and select the best targets is estimated to significantly impact the development of new drugs^10^. This is especially true for disease-modifying therapies^6,11^.

In recent years, various genome-wide association studies (GWAS) have identified over 100 loci associated with PD^12–18^. The largest European-ancestry PD GWAS identified 90 independent significant risk signals across 78 genomic regions (37,688 cases, 18,618 first-degree relatives of cases, and 1,417,791 controls)^13^. A polygenic risk score including 1,805 variants explained 16–36% of the heritable risk of PD^13^. Additionally, 15 population-specific variants were identified through GWAS conducted in individuals of Latino, East Asian, South Asian, and African and African admixed ancestry^14–17^. A recent large-scale multi-ancestry meta-analysis of PD GWAS uncovered 12 potentially novel loci and fine-mapped six putative causal variants at six previously identified loci (49,049 cases and 2,452,961 controls from these four ancestral populations)^18^.

A major limitation of GWAS is that it nominates genomic regions, not the specific genes that confer risk. Typically, GWAS hits can be explained by two general mechanisms: 1) a functional coding variant that alters protein function or 2) a non-coding variant that regulates transcription or translation. Often GWAS studies annotate their loci with the closest gene to the lead variant. This is the correct causal gene strikingly often^19^, although more sophisticated methods have been developed to improve predictive accuracy.

There have been previous gene prioritization efforts using machine learning models that were trained on and applied to non-PD traits (“locus-to-gene” (L2G) model^20^) or PD specifically (referred to as Yu2024^21^). However, all previous efforts to prioritize PD GWAS genes have done so on a locus-by-locus basis, ignoring information from other significant loci and the rest of the genome. The polygenic priority score (PoPS)^19^ is a gene prioritization tool that incorporates genome-wide information from MAGMA^22^ gene-level association tests and more than 57,000 gene-level features (*i.e*., gene expression, biological pathways, and protein-protein interactions). By intersecting genes with the top PoPS value in their locus with genes that were nearest to their GWAS signal, they were able to achieve a precision of 79%.

Here we applied PoPS to several PD GWAS summary statistics including European and East Asian ancestries and highlight high-confidence prioritized genes.

## Methods and Materials

### Ethics statement

This study was conducted in accordance with the ethical standards of the institutional and national research committees. Details on Institutional Review Board approvals of the individual studies included in the presented work are provided in the original publications^13,16^.

### GWAS summary statistics

We analyzed two published GWAS datasets. First, an East Asian-ancestry (EAS) meta-analysis of 6,724 cases and 24,851 controls (effective sample size [*N*_eff_] = 15,886)^16^. Second, a European-ancestry (EUR) meta-analysis of 37,688 cases, 18,618 proxy cases (first degree relative with PD), and 1,417,791 controls (*N*_eff_ = 127,626)^13^. We performed a fixed-effects meta-analysis of these two datasets using METAL^23^ to generate a combined EAS+EUR dataset.

### Reference panels

We used all available data from the Haplotype Reference Consortium release 1.1 (HRC) to construct three linkage disequilibrium (LD) reference panels: an EAS panel (*N* = 538), an EUR panel (*N* = 16,860), and an EAS+EUR panel that included both EAS and EUR individuals in the same proportions as the GWAS summary statistics - 11% EAS and 89% EUR (*N*_EUR_ = 4,322, *N*_EAS_ = 538).

### Variant quality control

We removed EUR GWAS variants with: 1) a reported allele frequency that differed from the EUR reference panel frequency by > 0.1 (42 variants removed) and 2) a reported allele frequency that differed from the EUR reference panel frequency by > 12-fold (an additional 462 variants removed). No variants failed these same quality control checks for the EAS GWAS and EAS+EUR meta-analysis. After quality control, the number of variants remaining was 5,347,472 for EAS, 6,584,031 for EUR, and 7,593,632 for EAS+EUR.

### Isolating independent association signals

In order to disentangle statistically-independent genetic signals in the EAS+EUR dataset, we first clumped variants using PLINK v1.9^24^ (*P* < 5×10^−8^, *r*^2^ < 0.1, window size = 3 Mbp) and our EAS+EUR reference panel, expanded the boundaries of each clump by 500kb on either side, and merged overlapping boundaries. Within each resulting region, we ran COJO^25^ and removed hits with joint *P* > 5×10^−8^. If multiple independent hits in a region were found, we used COJO to isolate each signal by performing leave-one-hit-out conditional analysis. For each isolated signal, we computed credible sets (CSs) using the finemap.abf function in the coloc R package^26,27^. Finally, we defined loci as ± 300 kb around each credible set.

### MAGMA and PoPS

We performed gene-based association tests using MAGMA^22^ (“SNP-wise mean model”) and all variants with MAF > 1%. We analyzed the EAS- and EUR-based GWAS separately using the corresponding ancestry-specific reference panel and MAFs. We mapped variants to protein-coding genes using Genome Reference Consortium Human Build 37 (GRCh37) gene start and end positions from GENCODE v44^28^. We removed genes that had fewer than three variants mapped to them. For each gene, we meta-analyzed the resulting ancestry-specific MAGMA z-scores weighted by the square root of sample size^23^. Using the ancestry-specific MAGMA results as input, we performed PoPS^19^ using all 57,543 gene-based features as predictors. These features were not available for chrX so we restricted our analysis to autosomal genes. The resulting ancestry-specific PoPS values were then also meta-analyzed weighted by the square root of sample size. We only used the meta-analyzed MAGMA and PoPS values for gene prioritization.

### Gene prioritization criteria

Following the original PoPS publication, we prioritized genes that met both of the following criteria: 1) had the top PoPS value in a given locus and 2) were the nearest gene to the corresponding GWAS signal based on the posterior inclusion probability (PIP)-weighted average position of credible set variants (the smallest set of variants expected to contain the causal variant with 95% probability). We also prioritized genes that had PIP > 50% for non-synonymous credible set variants affecting the gene. We used non-synonymous variants from the “baseline-LF 2.2.UKB model” (80,693 variants)^29^. If a locus contained multiple prioritized genes, we only included the gene that was prioritized due to non-synonymous credible set variants.

### Comparison with previous PD gene prioritization efforts

We compared our prioritized genes with those highlighted in two previous studies: Mountjoy *et al*. 2021^20^ and Yu *et al*. 2024^21^. Both studies trained XGBoost models to predict the probability that a given gene is causal in a given GWAS locus, and have been applied to the EUR PD GWAS data. We downloaded results for the Mountjoy *et al*. 2021 study^20^ from the Open Targets Genetics website (https://genetics.opentargets.org/Study/GCST009325/). We extracted results for the Yu *et al*. 2024^21^ study from Supplementary Table 2. Although there only appears to be one GWAS hit near the *FCGR2A* gene in the EUR dataset^13^, Yu *et al*. 2024^21^ causal probabilities in this locus sum to over 2500%. If the sum of Yu *et al*. 2024^21^ causal probabilities within a given locus exceeded 100%, we therefore rescaled them to sum to 100%.

### Drug repurposing and tractability

We determined whether our prioritized genes were targeted by approved or investigational drugs using GraphQL API queries of the Open Targets platform^30^, which in turn queries the EMBL-EBI ChEMBL database. For genes that were not targeted by approved or investigational drugs, we performed additional Open Targets API queries to extract evidence of drug tractability—the probability of identifying a small molecule drug that is able to bind and modulate a given target. We considered genes to encode proteins that may be druggable if they have been co-crystallized with a small molecule.

### Literature review

We performed a PubMed search (https://pubmed.ncbi.nlm.nih.gov/) for each prioritized gene using the standardized search term: “GENE_NAME[Title/Abstract] AND parkinson[Title/Abstract]”. We excluded known monogenic or high-risk genes: *LRRK2* (OMIM: 607060)*, GBA1* (OMIM: 168600)*, SNCA* (OMIM: 168601), and *VPS13C* (OMIM: 616840). We also excluded *TMEM175*, which has been experimentally confirmed as the causal gene in its locus. For each search that returned at least eight publications, we performed a more detailed literature review. We excluded genes with fewer than eight publications from further analyses. We screened the abstracts for evidence of a potential role of each gene in the pathogenesis of PD and information regarding the potential mechanism of action, thereby nominating it as a potential drug target. If required, an additional full-text review was performed.

## Results

### Methods overview

**Figure 1.**
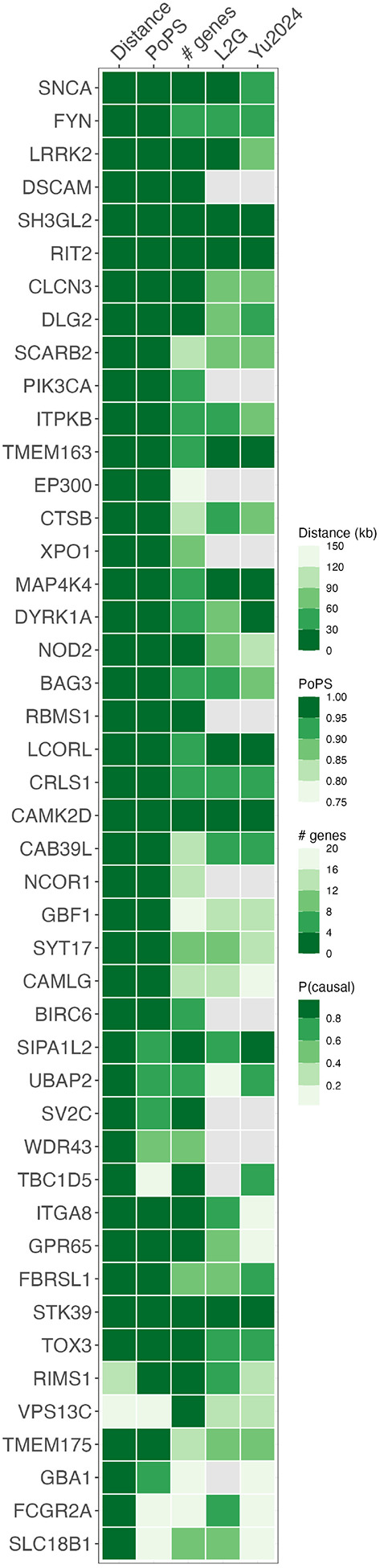
Heatmap of prioritized Parkinson’s disease genes. An overview of the evidence supporting each prioritized gene. Distance: distance in kilobases between gene and credible set. PoPS: PoPS percentile where 0 represents the smallest genome-wide value and 1 represents the largest. MAGMA: # genes: number of genes in the locus. L2G: Probability of being the causal genes according to the L2G model^20^. Yu2024: Probability of being the causal genes according to the Yu2024 model^21^.

We prioritized PD genes using a meta-analysis of the largest published GWASes in East Asian^16^ and European-ancestry^13^ individuals (44,412 cases, 18,618 proxy cases, and 1,442,642 controls). We identified 120 independent associations with *P* < 5×10^−8^ (Supplementary Table 1). Across these loci, we prioritized 46 PD genes (Fig. 1, Supplementary Table 2) based on their PoPS scores, distance to the credible set, and presence of non-synonymous variants in the credible set. We further compared our findings with prioritization efforts using the L2G model^20^ and from Yu *et al*. 2024^21^ (Fig. 1).

### Many prioritized genes are also supported by previous literature

Our analysis prioritized several known monogenic or high-risk PD genes, including *SNCA* (OMIM: 168601), *LRRK2* (OMIM: 607060), *GBA1* (OMIM: 168600), and *VPS13C* (OMIM: 616840). For some of these, PD clinical trials are already ongoing. *LRRK2*, thought to cause PD through a gain-of-function mechanism leading to increased LRRK2 kinase activity, is being targeted by kinase inhibitors^5^. In contrast, other therapies aim to activate glucocerebrosidase (GCase), encoded by *GBA1*, and increase its activity^31,32^. Furthermore, there are different approaches of targeting α-synuclein (encoded by *SNCA*), *e.g*., by reducing extracellular α-synuclein (PASADENA^33^ and SPARK^34,35^ trials) or blocking misfolding^36^ or aggregation^37^. We also prioritized *TMEM175*, which has been experimentally validated to be the causal gene in its respective GWAS locus^38^.

We identified several genes that were also supported by our literature review. Among these, *RIT2, DYRK1A, BAG3*, and *SCARB2* were particularly promising, with strong evidence highlighting their involvement in PD pathogenesis, although notably, no focussed locus dissection efforts have been performed yet. *RIT2* is linked to neuroprotective pathways in dopaminergic neurons^39^. Reduced RIT2 expression increases neurodegeneration in various preclinical PD models^40–43^, while RIT2 overexpression rescues autophagy-lysosome deficits and reduces α-synuclein aggregation^40,42^. *DYRK1A* has been linked to several neurodegeneration pathways^44,45^. DYRK1A appears to regulate α-synuclein inclusion formation^46^ and directly phosphorylate (and thereby inhibit) parkin^47^. *BAG3* plays a crucial role in macroautophagy and the clearance of alpha-synuclein aggregates^48,49^. *BAG3* overexpression also suppresses neuroinflammation, decreasing the release of pro-inflammatory cytokines and contributing to mitigating neuronal damage in PD^50–52^. *SCARB2* is essential for trafficking GCase from the endoplasmic reticulum to lysosomes^53^. Deficiencies in *SCARB2* lead to reduced GCase activity and subsequent α-synuclein accumulation, contributing to neurotoxicity in dopaminergic neurons^53–57^. Biallelic variants in *SCARB2* cause progressive myoclonic epilepsy with or without renal failure.

Furthermore, we prioritized several genes without strong literature evidence but predicted to be the most likely gene responsible underlying the PD GWAS signal with a probability greater than 80% by L2G or Yu2024 (*SIPA1L2, SH3GL2*, *TMEM163*, *MAP4K4*, *LCORL*, *CAMK2D*, *STK39*)^20,21^ (Fig. 1).

### Prioritized genes that encode potentially druggable targets for PD

Of the 46 prioritized genes, six (*FYN, DYRK1A, NOD2, CTSB, SV2C, ITPKB)* were identified as promising drug targets for PD, each supported by at least eight PD-related publications in our literature review. Their potential as therapeutic targets was established based either on their suitability for repurposing existing drugs to target them in PD (Supplementary Table 3) or their promise for new drug development using the Open Targets platform.

FYN inhibitors have been approved to treat various cancers (e.g., chronic myeloid leukemia and acute lymphoblastic leukemia)^58,59^ and tested in clinical trials for Alzheimer disease^60–62^. *FYN* plays a significant role in neuroinflammation and protein aggregation^63–65^. Inhibition of FYN has shown promise in reducing these detrimental processes and alleviating L-dopa-induced dyskinesia by regulating the phosphorylation of the NMDA receptor^66–68^.

*DYRK1A* encodes a dual-specificity kinase with a wide range of functions and interaction partners^45^. Although no approved drug targeting *DYRK1A* exists for PD, a number of DYRK1A inhibitors have been investigated in the context of other neurological diseases^69–72^. DYRK1A seems to directly phosphorylate a-synuclein and thereby affect aggregate formation and cell death in immortalized hippocampal neurons and brain tissue samples from a MPTP-induced PD mouse model, whereas DYRK1A inhibition suppressed a-synuclein aggregation and reduced dopaminergic neuron apoptosis^46,73^.

*CTSB* encodes cathepsin B (catB), a lysosomal enzyme crucial for protein degradation and regulating autophagy. CatB knock-out or inhibition impairs lysosomal functions in dopaminergic neurons, leads to reduced GCase activity^74,75^, and promotes α-synuclein aggregation^74^. However, farnesyltransferase inhibitors enhance cathepsin transport and activity, thereby improving lysosomal function and α-synuclein clearance^76^. These inhibitors, already approved for Hutchinson-Gilford progeria syndrome^77^, could have potential for repurposing in PD.

Although there is an approved drug targeting *NOD2* for osteosarcoma^78^, it acts as an activator and induces a proinflammatory response. However, data suggest that *NOD2* inhibition and an anti-inflammatory response would be desired in PD. In an experimental mouse PD model induced by the neurotoxin 6-hydroxydopamine, NOD2 deficiency was associated with an attenuated inflammatory response and suggested to have protective effects against degeneration of dopaminergic neurons and neuronal death^79^.

*SV2C* encodes synaptic vesicle glycoprotein 2C and is involved in synaptic vesicular function. *SV2C* expression is enriched in the basal ganglia, particularly in dopaminergic neurons in the substantia nigra^80^, and is believed to be crucial for dopamine neuron function and homeostasis^81,82^. In a mouse model, *SV2C* knock-out resulted in reduced dopamine release in the dorsal striatum, disrupted α-synuclein expression, and mild motor deficits^82^, suggesting that enhancing *SV2C* function is beneficial for PD. Clinical trials targeting *SV2C* are currently ongoing for epilepsy, although it is unclear whether the drug is an activator or inhibitor. Should further preclinical testing find that it is an activator, this drug could potentially be considered for repurposing for PD.

*ITPKB* encodes a kinase involved in calcium homeostasis^83^. While it is often possible to inhibit kinases, inhibiting ITPKB is predicted to be detrimental in PD since it has been associated with increased α-synuclein aggregation and impaired autophagy^84^. However, previous work has shown that *MICU3* (a brain-specific mitochondrial calcium uniporter) operates downstream of ITPKB and inhibiting MICU3 was able to rescue increased ITPKB activity^85^. *MICU3*, has the highest PoPS score in one of our GWAS loci, but was not the nearest gene and was therefore not prioritized (Supplementary Fig. 1).

**Figure 2.**
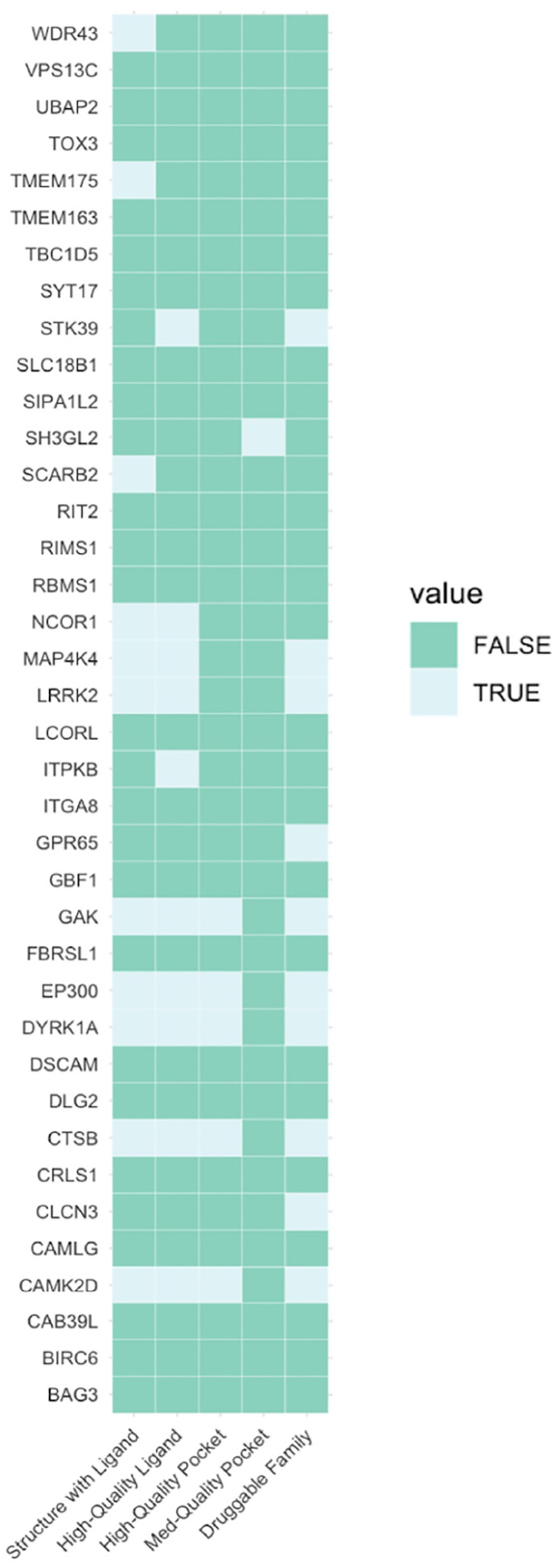
Small molecule target tractability assessment. Predicted tractability of the 38 prioritized genes that are not already targets of approved or investigational drugs. Data was extracted from the Open Targets platform using GraphQL API queries (https://platform.opentargets.org/) (see Methods). Various forms of evidence that suggest that a target may be tractable are shown on the x-axis, sorted from highest quality to lowest. Structure with Ligand: a Protein Data Bank co-crystal structure exists for the target and a small molecule. High-Quality Ligand: the target is bound by a ligand that 1) has a property forecast index ≤ 7, 2) binds ≤ 2 distinct protein domains and motifs identified by SMART (Simple Modular Architecture Research Tool), and 3) is derived from ≥ 2 distinct chemical scaffolds. High-Quality Pocket: the target has a DrugEBIlity score ≥ 0.7. Med-Quality Pocket: the target has a DrugEBIlity score 0–0.7. Druggable Family: the target was reported to be a member of the druggable genome in Finan *et al*. 2017^91^. Light green cells indicate that a given gene is supported by a given form of evidence, while dark green cells indicate an absence of such evidence. For more information on ongoing targeted drug trials for a selection of genes, see Supplementary Table 3.

Finally, we prioritized several genes with limited existing literature or functional studies pertaining to PD. Despite this, many of these genes have promising potential as drug targets for PD, *e.g*., by repurposing existing drugs approved for other conditions (Supplementary Table 3). For example, inhibitors of exportin 1 (encoded by *XPO1*), a nuclear export protein crucial for maintaining cellular homeostasis, have been developed and approved for different cancer conditions^86^. *PIK3CA* encodes a lipid kinase essential for cell proliferation and survival and has an approved inhibitor for breast cancer^87^. We identified five additional genes (*EP300, MAP4K4, CAMK2D, NCOR1*, and *WDR43*) that may encode druggable proteins (Fig. 2). Among those, *MAP4K4* and *CAMK2D* were also prioritized by the L2G^20^ and Yu2024^21^ models (Fig. 1). Further, *EP300* is not only the nearest gene with the top local PoPS score, but there is also a non-synonymous variant (rs20551) in the credible set in high LD (R^2^ = 87%) with the lead variant (rs9611522).

## Discussion

The field of targeted therapies driven by genetic discoveries for PD is rapidly evolving, with new therapies entering trials and ongoing studies bearing hope for more effective treatments. The goal of our study was to nominate high-confidence genes that warrant further follow-up investigations but bear promising potential as novel drug targets for PD. Following the established monogenic genes with ongoing trials already, *e.g., GBA1*, *LRRK2*, and *SNCA*, our prioritized genes may have the next-strongest links to PD and may represent our next-best opportunities for disease-modifying drug targets.

We prioritized 46 genes predicted to play a potentially important role in PD. In addition to prioritizing known and well-established PD genes, including *SNCA*, *LRRK2*, *GBA1*, *TMEM175* and *VPS13C*, we identified six genes (*FYN, DYRK1A, NOD2, CTSB, SV2C*, and *ITPKB*) emerged as promising targets for drug repurposing or novel therapeutic development. Our literature review found that each of these genes had a plausible mechanistic link to PD pathogenesis, paving the way for additional preclinical replication and validation experiments. We also nominated *XPO1, PIK3CA, EP300, MAP4K4, CAMK2D, NCOR1*, and *WDR43*. Despite limited prior research, these genes may be druggable and warrant further investigation. Lastly, we identified genes such as *RIT2, BAG3*, and *SCARB2*, with strong literature evidence supporting their involvement in neurodegenerative pathways linked to PD.

### The strength of PoPS compared to previous gene prioritization efforts

Several previous gene prioritization efforts have aimed to identify the causal genes in PD GWAS loci. For example, the PD GWAS browser is an online tool that displays data from a variety of resources for relevant PD risk loci, including GWAS statistics, eQTL, burden, expression, constraint, and literature data^88^. It serves as an exploratory scoring system that can be configured by users to assist them with the prioritization of genes located within PD GWAS loci. OmicSynth is a resource leveraging large-scale genetic and genomic data in a Mendelian randomization framework to identify therapeutic targets for neurodegenerative diseases^89^.

The “locus-to-gene” (L2G) model is an XGBoost model trained on data from 445 curated gold-standard GWAS loci from 194 traits^20^. L2G integrates fine-mapping with functional genomics data, gene distance, and *in silico* functional predictions. The model has been applied to a large number of GWAS from a variety of traits, including the largest European-ancestry PD GWAS^13^. A similar but PD-specific XGBoost model was developed by Yu and colleagues (Yu2024)^21^. They trained their model on the largest European-ancestry PD GWAS^13^, using only seven well-established PD-associated genes as positive labels: *GBA1, LRRK2, SNCA, GCH1, MAPT, TMEM175*, and *VPS13C*. They used predictive features derived from gene expression and splicing in PD-relevant tissues such as brain, dopaminergic neuron subpopulations, and microglia.

The main advantage of the Yu2024 model over the L2G model is its PD-centred design. However, predictions made by the Yu2024 model appear to be poorly calibrated, possibly due to the small number of positive labels in its training set. For example, the largest European-ancestry PD GWAS^13^ identified a GWAS hit in strong linkage disequilibrium (LD, r^2^ = 97.9%) with a non-synonymous variant in the *FCGR2A* gene, strongly suggesting that this is the causal gene in the locus. Although conditional analyses revealed that this was the only independent signal in the locus, the Yu2024 model predicted that 19 genes in this locus had > 75% probability of being the causal gene with *FCGR2A* only ranking 18th.

All these previous efforts used a locus-by-locus approach, disregarding data from other GWAS loci and the remainder of the genome. In comparison, PoPS incorporates genome-wide information. The initial PoPS publication^19^ shows that PoPS outperformed other similarity-based and locus-based gene prioritization methods across numerous complex traits. By combining PoPS with nearest gene and non-synonymous credible set variant information, we prioritized a list of high-confidence genes as promising candidates for follow-up functional studies and potential drug development programs.

### Prioritization of genes with strong evidence supporting a mechanistic link to PD

Our work identified 46 genes that are very likely to be driving their signal in their respective GWAS loci. For a subset of these genes, there is also substantial literature supporting a mechanistic connection to PD. Some of these (*FYN*, *DYRK1A*, *NOD2*, *CTSB*, *SV2C*, and *ITPKB*) may serve as potential PD drug targets due to their involvement in pathways linked to neuroinflammation, autophagy, and neuronal degeneration. However, extensive further preclinical work is required to determine if and how these genes can be targeted and how this research can be translated into potential drug development. Another strategy for some of these genes is to evaluate how already existing drugs for other conditions may be repurposed for PD. Together, we hope these results stimulate interest in initiating drug development programs focused on these targets, all of which represent promising candidates for future PD therapies.

Approved FYN inhibitors could potentially be repurposed to reduce neuroinflammation and protein aggregation in PD patients^63–65^. Approved farnesyltransferase inhibitors could potentially be repurposed to enhance cathepsin activity, lysosomal function, and α-synuclein clearance^76,77^. *DYRK1A* inhibition may be a promising approach to investigate a potential reduction of α-synuclein aggregation and neuronal apoptosis. By developing MICU3 inhibitors, it may be possible to improve autophagy and reduce α-synuclein aggregation driven by elevated *ITPKB* activity. Although an approved drug already targets *NOD2*, it appears to have proinflammatory effects. By developing NOD2 inhibitors, it may be possible to decrease neuroinflammation and protect against dopaminergic neuron degeneration^79^. Additional research is also needed to determine the mechanism of action of investigational epilepsy drugs targeting SV2C, as they could potentially be repurposed for PD if confirmed as safe and effective activators.

### Prioritization of genes representing potentially druggable targets for PD

Furthermore, our approach identified a group of genes that has been relatively unexplored in PD. Our target tractability assessment indicated that some of these genes may be druggable targets, including *XPO1, PIK3CA, EP300, MAP4K4, CAMK2D, NCOR1*, and *WDR43*. Moreover, XPO1 and PIK3CA inhibitors, currently approved for the treatment of various cancer types, hold potential for repurposing as therapeutic agents for PD^86,87^. We hope this first piece of evidence will stimulate further investigations and advocate for studies exploring the potential effects of overexpression and knockdown of these genes in cell and animal models for PD to better understand their potential mechanistic role. Such extensive preclinical work is crucial to facilitating and advancing drug development programs by pharmaceutical companies, and represents a critical step toward establishing these genes as viable therapeutic targets for PD.

### Limitations

Our study had several limitations. We were unable to assess genes on chromosome X because PoPS gene features are restricted to autosomes. Our approach failed to prioritize *GCH1*, a well-established PD risk gene^13,90^ implicated in dopamine synthesis in nigrostriatal cells. Although *GCH1* had the top PoPS value in its locus, the PIP-weighted centre of the credible set was slightly closer to the *WDHD1* gene. Previous machine learning gene prioritization efforts identified four genes with probability > 80% of being a causal PD gene that were not prioritized by our methods: *KCNS3*, *MBNL2*, *ASXL3*, and *SCAF11*^21^. However, there is only one genome-wide significant variant in the *KCNS3* locus (gnomAD non-Finnish European MAF = 9.7%) and all other variant P values are greater than 1×10^−5^, suggesting this may be a spurious association. *MBNL2*, *ASXL3*, and *SCAF11* were all the nearest gene in their loci, but did not have the top PoPS value.

Using GWAS data only from European and East Asian- ancestry could potentially restrict the generalizability of our findings across diverse genetic populations. The limited representation of these populations in GWAS highlights the need for larger studies in underrepresented groups to ensure equitable insights into PD genetics. Initiatives like the Global Parkinson’s Genetics Program (GP2) have been pivotal in addressing this gap, dedicating considerable efforts to advancing the study of underrepresented populations and fostering global collaborations in PD research.

### Conclusion

Gene prioritization efforts play a crucial role in nominating the underlying causal genes within GWAS risk loci, enabling the identification of potential novel drug targets. These efforts are essential for translating genetic discoveries into actionable therapies and have paved the way for personalized medicine approaches. Using PoPS and other methods, we prioritized a high-confidence list of 46 genes that are predicted to play an important role in PD pathogenesis and represent potential therapeutic targets. Prioritizing known and well-established PD genes, already targeted in clinical drug trials, strengthens the robustness and reliability of our approach. Newly prioritized genes may represent our next-best candidates for disease-modifying therapeutics. We hope our findings stimulate further investigations and preclinical work to facilitate drug development programs and potentially establish these genes as viable therapeutic targets for PD.

## Supplementary Material

Supplement 1

Supplement 2 **Figure S1. Variant-level associations and PoPS results for the *MICU3* locus.** The upper portion of each sub-plot is a LocusZoom plot. Each point represents a different genetic variant, the x-axis represents physical position on the listed chromosome, the left y-axis represents −log_10_-transformed P value, the right y-axis represents the recombination rate, colour represents linkage disequilibrium with the lead variant in the locus (as shown in the legend), and the horizontal dashed line represents the genome-wide significance P value threshold of 5×10^−8^. The lower portion of each figure is a PoPS plot. Genes are denoted as blue bars spanning from their transcription start site to their transcription stop site using the same x-axis as the LocusZoom plot, the y-axis represents the raw PoPS score, the dashed horizontal grey lines represent the top 10% and 1% of PoPS scores genome-wide, and the solid horizontal grey line represents a PoPS score of 0.

## Figures and Tables

**Figure 3.**
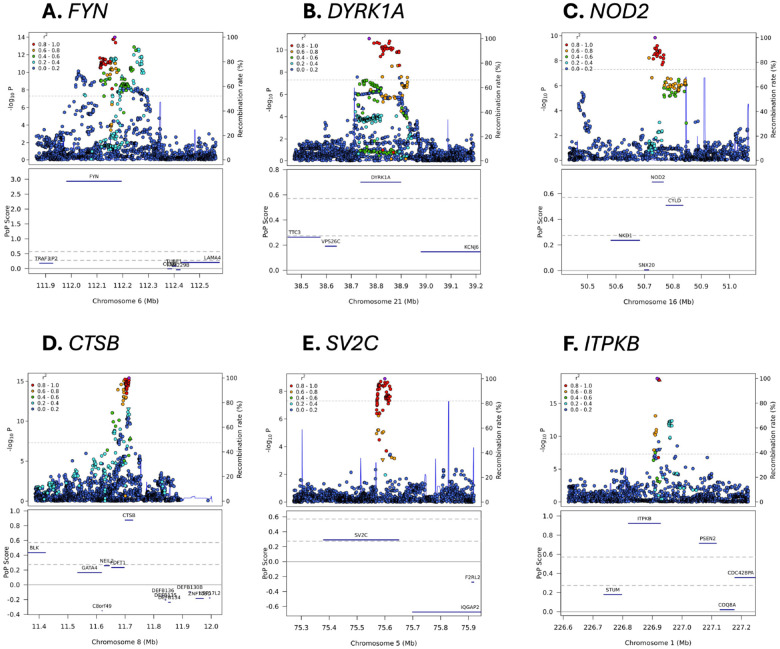
Variant-level associations and PoPS results for selected loci. The upper portion of each sub-plot is a LocusZoom plot. Each point represents a different genetic variant, the x-axis represents physical position on the listed chromosome, the left y-axis represents −log_10_-transformed P value, the right y-axis represents the recombination rate, colour represents linkage disequilibrium with the lead variant in the locus (as shown in the legend), and the horizontal dashed line represents the genome-wide significance P value threshold of 5×10^−8^. The lower portion of each figure is a PoPS plot. Genes are denoted as blue bars spanning from their transcription start site to their transcription stop site using the same x-axis as the LocusZoom plot, the y-axis represents the raw PoPS score, the dashed horizontal grey lines represent the top 10% and 1% of PoPS scores genome-wide, and the solid horizontal grey line represents a PoPS score of 0.

## Data Availability

ChEMBL Database: https://www.ebi.ac.uk/chembl/ HRC reference release 1.1: https://ega-archive.org/datasets/EGAD00001002729 Gencode release 44: https://www.gencodegenes.org/human/release_44.html OpenTargets platform: https://platform-docs.opentargets.org/ Details for accessing the EUR^13^ and EAS^16^ GWAS datasets can be found their original publications.

## References

[1] GBD 2016 Parkinson’s Disease Collaborators. Global, regional, and national burden of Parkinson’s disease, 1990–2016: a systematic analysis for the Global Burden of Disease Study 2016. Lancet Neurol. 2018;17(11):939–953. 30287051 10.1016/S1474-4422(18)30295-3 PMC6191528

[2] Periñán MT, Brolin K, Bandres-Ciga S, Effect Modification between Genes and Environment and Parkinson’s Disease Risk. Ann Neurol. 2022;92(5):715–724. 35913124 10.1002/ana.26467 PMC9588606

[3] Neumann WJ, Horn A, Kühn AA. Insights and opportunities for deep brain stimulation as a brain circuit intervention. Trends Neurosci. 2023;46(6):472–487. 37105806 10.1016/j.tins.2023.03.009

[4] Davidson B, Milosevic L, Kondrataviciute L, Kalia LV, Kalia SK. Neuroscience fundamentals relevant to neuromodulation: Neurobiology of deep brain stimulation in Parkinson’s disease. Neurotherapeutics. 2024;21(3):e00348. 38579455 10.1016/j.neurot.2024.e00348 PMC11000190

[5] McFarthing K, Buff S, Rafaloff G, Parkinson’s Disease Drug Therapies in the Clinical Trial Pipeline: 2023 Update. J Parkinsons Dis. 2023;13(4):427–439. 37302040 10.3233/JPD-239901 PMC10357160

[6] Minikel EV, Painter JL, Dong CC, Nelson MR. Refining the impact of genetic evidence on clinical success. Nature. Published online April 17, 2024. doi:10.1038/s41586-024-07316-0 PMC11096124 38632401

[7] King EA, Davis JW, Degner JF. Are drug targets with genetic support twice as likely to be approved? Revised estimates of the impact of genetic support for drug mechanisms on the probability of drug approval. PLoS Genet. 2019;15(12):e1008489. 31830040 10.1371/journal.pgen.1008489 PMC6907751

[8] Ochoa D, Karim M, Ghoussaini M, Hulcoop DG, McDonagh EM, Dunham I. Human genetics evidence supports two-thirds of the 2021 FDA-approved drugs. Nat Rev Drug Discov. 2022;21(8):551. 35804044 10.1038/d41573-022-00120-3

[9] Rusina PV, Falaguera MJ, Romero JMR, McDonagh EM, Dunham I, Ochoa D. Genetic support for FDA-approved drugs over the past decade. Nat Rev Drug Discov. 2023;22(11):864. 37803084 10.1038/d41573-023-00158-x

[10] Nelson MR, Tipney H, Painter JL, The support of human genetic evidence for approved drug indications. Nat Genet. 2015;47(8):856–860. 26121088 10.1038/ng.3314

[11] Cully M. Genetic information adds supporting weight. Nat Rev Drug Discov. 2015;14(8):525–525. 26228753 10.1038/nrd4692

[12] Khani M, Cerquera-Cleves C, Kekenadze M, Wild Crea P, Singleton AB, Bandres-Ciga S. Towards a Global View of Parkinson’s Disease Genetics. Ann Neurol. 2024;95(5):831–842. 38557965 10.1002/ana.26905 PMC11060911

[13] Nalls MA, Blauwendraat C, Vallerga CL, Identification of novel risk loci, causal insights, and heritable risk for Parkinson’s disease: a meta-analysis of genome-wide association studies. Lancet Neurol. 2019;18(12):1091–1102. 31701892 10.1016/S1474-4422(19)30320-5 PMC8422160

[14] Loesch DP, Horimoto ARVR, Heilbron K, Characterizing the Genetic Architecture of Parkinson’s Disease in Latinos. Ann Neurol. 2021;90(3):353–365. 34227697 10.1002/ana.26153 PMC8457043

[15] Foo JN, Tan LC, Irwan ID, Genome-wide association study of Parkinson’s disease in East Asians. Hum Mol Genet. 2017;26(1):226–232. 28011712 10.1093/hmg/ddw379

[16] Foo JN, Chew EGY, Chung SJ, Identification of Risk Loci for Parkinson Disease in Asians and Comparison of Risk Between Asians and Europeans: A Genome-Wide Association Study. JAMA Neurol. 2020;77(6):746–754. 32310270 10.1001/jamaneurol.2020.0428 PMC7171584

[17] Rizig M, Bandres-Ciga S, Makarious MB, Identification of genetic risk loci and causal insights associated with Parkinson’s disease in African and African admixed populations: a genome-wide association study. Lancet Neurol. 2023;22(11):1015–1025. 37633302 10.1016/S1474-4422(23)00283-1 PMC10593199

[18] Kim JJ, Vitale D, Otani DV, Multi-ancestry genome-wide association meta-analysis of Parkinson’s disease. Nat Genet. 2024;56(1):27–36. 38155330 10.1038/s41588-023-01584-8 PMC10786718

[19] Weeks EM, Ulirsch JC, Cheng NY, Leveraging polygenic enrichments of gene features to predict genes underlying complex traits and diseases. Nat Genet. 2023;55(8):1267–1276. 37443254 10.1038/s41588-023-01443-6 PMC10836580

[20] Mountjoy E, Schmidt EM, Carmona M, An open approach to systematically prioritize causal variants and genes at all published human GWAS trait-associated loci. Nat Genet. 2021;53(11):1527–1533. 34711957 10.1038/s41588-021-00945-5 PMC7611956

[21] Yu E, Larivière R, Thomas RA, Machine learning nominates the inositol pathway and novel genes in Parkinson’s disease. Brain. 2024;147(3):887–899. 37804111 10.1093/brain/awad345 PMC10907089

[22] de Leeuw CA, Mooij JM, Heskes T, Posthuma D. MAGMA: generalized gene-set analysis of GWAS data. PLoS Comput Biol. 2015;11(4):e1004219. 25885710 10.1371/journal.pcbi.1004219 PMC4401657

[23] Willer CJ, Li Y, Abecasis GR. METAL: fast and efficient meta-analysis of genomewide association scans. Bioinformatics. 2010;26(17):2190–2191. 20616382 10.1093/bioinformatics/btq340 PMC2922887

[24] Chang CC, Chow CC, Tellier LC, Vattikuti S, Purcell SM, Lee JJ. Second-generation PLINK: rising to the challenge of larger and richer datasets. Gigascience. 2015;4:7. 25722852 10.1186/s13742-015-0047-8 PMC4342193

[25] Yang J, Ferreira T, Morris AP, Conditional and joint multiple-SNP analysis of GWAS summary statistics identifies additional variants influencing complex traits. Nat Genet. 2012;44(4):369–375, S1–S3. 22426310 10.1038/ng.2213 PMC3593158

[26] Giambartolomei C, Vukcevic D, Schadt EE, Bayesian test for colocalisation between pairs of genetic association studies using summary statistics. PLoS Genet. 2014;10(5):e1004383. 24830394 10.1371/journal.pgen.1004383 PMC4022491

[27] Wallace C. Eliciting priors and relaxing the single causal variant assumption in colocalisation analyses. PLoS Genet. 2020;16(4):e1008720. 32310995 10.1371/journal.pgen.1008720 PMC7192519

[28] Frankish A, Diekhans M, Ferreira AM, GENCODE reference annotation for the human and mouse genomes. Nucleic Acids Res. 2019;47(D1):D766–D773. 30357393 10.1093/nar/gky955 PMC6323946

[29] Weissbrod O, Hormozdiari F, Benner C, Functionally informed fine-mapping and polygenic localization of complex trait heritability. Nat Genet. 2020;52(12):1355–1363. 33199916 10.1038/s41588-020-00735-5 PMC7710571

[30] Koscielny G, An P, Carvalho-Silva D, Open Targets: a platform for therapeutic target identification and validation. Nucleic Acids Res. 2017;45(D1):D985–D994. 27899665 10.1093/nar/gkw1055 PMC5210543

[31] Giladi N, Alcalay RN, Cutter G, Safety and efficacy of venglustat in GBA1-associated Parkinson’s disease: an international, multicentre, double-blind, randomised, placebo-controlled, phase 2 trial. Lancet Neurol. 2023;22(8):661–671. 37479372 10.1016/S1474-4422(23)00205-3

[32] den Heijer JM, Kruithof AC, Moerland M, A Phase 1B Trial in GBA1-Associated Parkinson’s Disease of BIA-28–6156, a Glucocerebrosidase Activator. Mov Disord. 2023;38(7):1197–1208. 37195859 10.1002/mds.29346

[33] Pagano G, Boess FG, Taylor KI, A Phase II Study to Evaluate the Safety and Efficacy of Prasinezumab in Early Parkinson’s Disease (PASADENA): Rationale, Design, and Baseline Data. Front Neurol. 2021;12:705407. 34659081 10.3389/fneur.2021.705407 PMC8518716

[34] Brys M, Fanning L, Hung S, Randomized phase I clinical trial of anti-α-synuclein antibody BIIB054. Mov Disord. 2019;34(8):1154–1163. 31211448 10.1002/mds.27738 PMC6771554

[35] Lang AE, Siderowf AD, Macklin EA, Trial of Cinpanemab in Early Parkinson’s Disease. N Engl J Med. 2022;387(5):408–420. 35921450 10.1056/NEJMoa2203395

[36] Price DL, Koike MA, Khan A, The small molecule alpha-synuclein misfolding inhibitor, NPT200–11, produces multiple benefits in an animal model of Parkinson’s disease. Sci Rep. 2018;8(1):16165. 30385782 10.1038/s41598-018-34490-9 PMC6212487

[37] Pagan FL, Hebron ML, Wilmarth B, Nilotinib Effects on Safety, Tolerability, and Potential Biomarkers in Parkinson Disease: A Phase 2 Randomized Clinical Trial. JAMA Neurol. 2020;77(3):309–317. 31841599 10.1001/jamaneurol.2019.4200 PMC6990742

[38] Jinn S, Blauwendraat C, Toolan D, Functionalization of the TMEM175 p.M393T variant as a risk factor for Parkinson disease. Hum Mol Genet. 2019;28(19):3244–3254. 31261387 10.1093/hmg/ddz136 PMC6859430

[39] Uenaka T, Satake W, Cha PC, In silico drug screening by using genome-wide association study data repurposed dabrafenib, an anti-melanoma drug, for Parkinson’s disease. Hum Mol Genet. 2018;27(22):3974–3985. 30137437 10.1093/hmg/ddy279 PMC6216208

[40] Obergasteiger J, Castonguay AM, Pizzi S, The small GTPase Rit2 modulates LRRK2 kinase activity, is required for lysosomal function and protects against alpha-synuclein neuropathology. NPJ Parkinsons Dis. 2023;9(1):44. 36973269 10.1038/s41531-023-00484-2 PMC10042831

[41] Kearney PJ, Zhang Y, Liang M, Silencing Parkinson’s risk allele Rit2 sex-specifically compromises motor function and dopamine neuron viability. NPJ Parkinsons Dis. 2024;10(1):41. 38395968 10.1038/s41531-024-00648-8 PMC10891080

[42] Olsen AL, Feany MB. Parkinson’s disease risk genes act in glia to control neuronal α-synuclein toxicity. Neurobiol Dis. 2021;159:105482. 34390834 10.1016/j.nbd.2021.105482 PMC8502212

[43] Wang Q, Wang M, Choi I, Molecular profiling of human substantia nigra identifies diverse neuron types associated with vulnerability in Parkinson’s disease. Sci Adv. 2024;10(2):eadi8287. 38198537 10.1126/sciadv.adi8287 PMC10780895

[44] Demuro S, Di Martino RMC, Ortega JA, Cavalli A. GSK-3β, FYN, and DYRK1A: Master Regulators in Neurodegenerative Pathways. Int J Mol Sci. 2021;22(16). doi:10.3390/ijms22169098 PMC8396491 34445804

[45] Arbones ML, Thomazeau A, Nakano-Kobayashi A, Hagiwara M, Delabar JM. DYRK1A and cognition: A lifelong relationship. Pharmacol Ther. 2019;194:199–221. 30268771 10.1016/j.pharmthera.2018.09.010

[46] Kim EJ, Sung JY, Lee HJ, Dyrk1A phosphorylates alpha-synuclein and enhances intracellular inclusion formation. J Biol Chem. 2006;281(44):33250–33257. 16959772 10.1074/jbc.M606147200

[47] Im E, Chung KC. Dyrk1A phosphorylates parkin at Ser-131 and negatively regulates its ubiquitin E3 ligase activity. J Neurochem. 2015;134(4):756–768. 25963095 10.1111/jnc.13164

[48] Cao YL, Yang YP, Mao CJ, A role of BAG3 in regulating SNCA/α-synuclein clearance via selective macroautophagy. Neurobiol Aging. 2017;60:104–115. 28941726 10.1016/j.neurobiolaging.2017.08.023

[49] Seidel K, Vinet J, Dunnen WFA den, The HSPB8-BAG3 chaperone complex is upregulated in astrocytes in the human brain affected by protein aggregation diseases. Neuropathol Appl Neurobiol. 2012;38(1):39–53. 21696420 10.1111/j.1365-2990.2011.01198.x

[50] Sheehan PW, Nadarajah CJ, Kanan MF, An astrocyte BMAL1-BAG3 axis protects against alpha-synuclein and tau pathology. Neuron. 2023;111(15):2383–2398.e7. 37315555 10.1016/j.neuron.2023.05.006 PMC10524543

[51] Lu SZ, Guo YS, Liang PZ, Suppression of astrocytic autophagy by αB-crystallin contributes to α-synuclein inclusion formation. Transl Neurodegener. 2019;8:3. 30675347 10.1186/s40035-018-0143-7 PMC6337871

[52] Ying ZM, Lv QK, Yao XY, BAG3 promotes autophagy and suppresses NLRP3 inflammasome activation in Parkinson’s disease. Ann Transl Med. 2022;10(22):1218. 36544667 10.21037/atm-22-5159 PMC9761134

[53] Keo A, Mahfouz A, Ingrassia AMT, Transcriptomic signatures of brain regional vulnerability to Parkinson’s disease. Commun Biol. 2020;3(1):101. 32139796 10.1038/s42003-020-0804-9 PMC7058608

[54] Thomas R, Moloney EB, Macbain ZK, Hallett PJ, Isacson O. Fibroblasts from idiopathic Parkinson’s disease exhibit deficiency of lysosomal glucocerebrosidase activity associated with reduced levels of the trafficking receptor LIMP2. Mol Brain. 2021;14(1):16. 33468204 10.1186/s13041-020-00712-3 PMC7816505

[55] Pérez-Roca L, Prada-Dacasa P, Segú-Vergés C, Glucocerebrosidase regulators SCARB2 and TFEB are up-regulated in Lewy body disease brain. Neurosci Lett. 2019;706:164–168. 31116970 10.1016/j.neulet.2019.05.034

[56] Zunke F, Andresen L, Wesseler S, Characterization of the complex formed by β-glucocerebrosidase and the lysosomal integral membrane protein type-2. Proc Natl Acad Sci U S A. 2016;113(14):3791–3796. 27001828 10.1073/pnas.1514005113 PMC4833265

[57] Ambrosi G, Ghezzi C, Zangaglia R, Levandis G, Pacchetti C, Blandini F. Ambroxol-induced rescue of defective glucocerebrosidase is associated with increased LIMP-2 and saposin C levels in GBA1 mutant Parkinson’s disease cells. Neurobiol Dis. 2015;82:235–242. 26094596 10.1016/j.nbd.2015.06.008

[58] Kohal R, Bhavana Kumari P, Sharma AK, Gupta GD, Verma SK. Fyn, Blk, and Lyn kinase inhibitors: A mini-review on medicinal attributes, research progress, and future insights. Bioorg Med Chem Lett. 2024;102:129674. 38408513 10.1016/j.bmcl.2024.129674

[59] Peng S, Fu Y. FYN: emerging biological roles and potential therapeutic targets in cancer. J Transl Med. 2023;21(1):84. 36740671 10.1186/s12967-023-03930-0 PMC9901160

[60] Ramos R, Vale N. Dual Drug Repurposing: The Example of Saracatinib. Int J Mol Sci. 2024;25(8). doi:10.3390/ijms25084565 PMC11050334 38674150

[61] Nygaard HB, Wagner AF, Bowen GS, A phase Ib multiple ascending dose study of the safety, tolerability, and central nervous system availability of AZD0530 (saracatinib) in Alzheimer’s disease. Alzheimers Res Ther. 2015;7(1):35. 25874001 10.1186/s13195-015-0119-0 PMC4396171

[62] van Dyck CH, Nygaard HB, Chen K, Effect of AZD0530 on Cerebral Metabolic Decline in Alzheimer Disease: A Randomized Clinical Trial. JAMA Neurol. 2019;76(10):1219–1229. 31329216 10.1001/jamaneurol.2019.2050 PMC6646979

[63] Panicker N, Saminathan H, Jin H, Fyn Kinase Regulates Microglial Neuroinflammatory Responses in Cell Culture and Animal Models of Parkinson’s Disease. J Neurosci. 2015;35(27):10058–10077. 26157004 10.1523/JNEUROSCI.0302-15.2015 PMC4495236

[64] Panicker N, Sarkar S, Harischandra DS, Fyn kinase regulates misfolded α-synuclein uptake and NLRP3 inflammasome activation in microglia. J Exp Med. 2019;216(6):1411–1430. 31036561 10.1084/jem.20182191 PMC6547864

[65] Low CYB, Lee JH, Lim FTW, Isoform-specific upregulation of FynT kinase expression is associated with tauopathy and glial activation in Alzheimer’s disease and Lewy body dementias. Brain Pathol. 2021;31(2):253–266. 33128789 10.1111/bpa.12917 PMC8017997

[66] Sanz-Blasco S, Bordone MP, Damianich A, The Kinase Fyn As a Novel Intermediate in L-DOPA-Induced Dyskinesia in Parkinson’s Disease. Mol Neurobiol. 2018;55(6):5125–5136. 28840468 10.1007/s12035-017-0748-3

[67] Saminathan H, Charli A, Luo J, Fyn kinase mediates pro-inflammatory response in a mouse model of endotoxemia: Relevance to translational research. Eur J Pharmacol. 2020;881:173259. 32565338 10.1016/j.ejphar.2020.173259 PMC7483871

[68] Saminathan H, Ghosh A, Zhang D, Fyn Kinase-Mediated PKCδ Y311 Phosphorylation Induces Dopaminergic Degeneration in Cell Culture and Animal Models: Implications for the Identification of a New Pharmacological Target for Parkinson’s Disease. Front Pharmacol. 2021;12:631375. 33995031 10.3389/fphar.2021.631375 PMC8113680

[69] Pathak A, Rohilla A, Gupta T, DYRK1A kinase inhibition with emphasis on neurodegeneration: A comprehensive evolution story-cum-perspective. Eur J Med Chem. 2018;158:559–592. 30243157 10.1016/j.ejmech.2018.08.093

[70] Nguyen TL, Fruit C, Hérault Y, Meijer L, Besson T. Dual-specificity tyrosine phosphorylationregulated kinase 1A (DYRK1A) inhibitors: a survey of recent patent literature. Expert Opin Ther Pat. 2017;27(11):1183–1199. 28766366 10.1080/13543776.2017.1360285

[71] Feki A, Hibaoui Y. DYRK1A Protein, A Promising Therapeutic Target to Improve Cognitive Deficits in Down Syndrome. Brain Sci. 2018;8(10). doi:10.3390/brainsci8100187 PMC6210095 30332747

[72] Jarhad DB, Mashelkar KK, Kim HR, Noh M, Jeong LS. Dual-Specificity Tyrosine Phosphorylation-Regulated Kinase 1A (DYRK1A) Inhibitors as Potential Therapeutics. J Med Chem. 2018;61(22):9791–9810. 29985601 10.1021/acs.jmedchem.8b00185

[73] Yong Y, Wu Q, Meng X, Dyrk1a phosphorylation of α-synuclein mediating apoptosis of dopaminergic neurons in Parkinson’s disease. Parkinsons Dis. 2023;2023:8848642. 37469393 10.1155/2023/8848642 PMC10352525

[74] Jones-Tabah J, He K, Senkevich K, The Parkinson’s disease risk gene cathepsin B promotes fibrillar alpha-synuclein clearance, lysosomal function and glucocerebrosidase activity in dopaminergic neurons. Res Sq. Published online March 19, 2024. doi:10.21203/rs.3.rs-3979098/v1 PMC11587650 39587654

[75] Kim MJ, Jeong H, Krainc D. Lysosomal ceramides regulate cathepsin B-mediated processing of saposin C and glucocerebrosidase activity. Hum Mol Genet. 2022;31(14):2424–2437. 35181782 10.1093/hmg/ddac047 PMC9307309

[76] Drobny A, Boros FA, Balta D, Reciprocal effects of alpha-synuclein aggregation and lysosomal homeostasis in synucleinopathy models. Transl Neurodegener. 2023;12(1):31. 37312133 10.1186/s40035-023-00363-z PMC10262594

[77] Mullard A. The FDA approves a first farnesyltransferase inhibitor. Nat Rev Drug Discov. 2021;20(1):8. 10.1038/d41573-020-00213-x 33277623

[78] Frampton JE. Mifamurtide: a review of its use in the treatment of osteosarcoma. Paediatr Drugs. 2010;12(3):141–153. 20481644 10.2165/11204910-000000000-00000

[79] Cheng L, Chen L, Wei X, NOD2 promotes dopaminergic degeneration regulated by NADPH oxidase 2 in 6-hydroxydopamine model of Parkinson’s disease. J Neuroinflammation. 2018;15(1):243. 30157869 10.1186/s12974-018-1289-z PMC6116377

[80] Dardou D, Dassesse D, Cuvelier L, Deprez T, De Ryck M, Schiffmann SN. Distribution of SV2C mRNA and protein expression in the mouse brain with a particular emphasis on the basal ganglia system. Brain Res. 2011;1367:130–145. 20869353 10.1016/j.brainres.2010.09.063

[81] Bucher ML, Dunn AR, Bradner JM, Synaptic vesicle glycoprotein 2C enhances vesicular storage of dopamine and counters dopaminergic toxicity. Eur J Neurosci. 2024;59(10):2483–2501. 38532289 10.1111/ejn.16311 PMC11647951

[82] Dunn AR, Stout KA, Ozawa M, Synaptic vesicle glycoprotein 2C (SV2C) modulates dopamine release and is disrupted in Parkinson disease. Proc Natl Acad Sci U S A. 2017;114(11):E2253–E2262. 28246328 10.1073/pnas.1616892114 PMC5358362

[83] Kovacs G, Reimer L, Jensen PH. Endoplasmic Reticulum-Based Calcium Dysfunctions in Synucleinopathies. Front Neurol. 2021;12:742625. 34744980 10.3389/fneur.2021.742625 PMC8563702

[84] Di Leva F, Filosi M, Oyston L, Increased Levels of the Parkinson’s Disease-Associated Gene ITPKB Correlate with Higher Expression Levels of α-Synuclein, Independent of Mutation Status. Int J Mol Sci. 2023;24(3). doi:10.3390/ijms24031984 PMC9916293 36768321

[85] Apicco DJ, Shlevkov E, Nezich CL, The Parkinson’s disease-associated gene ITPKB protects against α-synuclein aggregation by regulating ER-to-mitochondria calcium release. Proc Natl Acad Sci U S A. 2021;118(1). doi:10.1073/pnas.2006476118 PMC7817155 33443159

[86] Azmi AS, Uddin MH, Mohammad RM. The nuclear export protein XPO1 - from biology to targeted therapy. Nat Rev Clin Oncol. 2021;18(3):152–169. 33173198 10.1038/s41571-020-00442-4

[87] André F, Ciruelos E, Rubovszky G, Alpelisib for PIK3CA-Mutated, Hormone Receptor-Positive Advanced Breast Cancer. N Engl J Med. 2019;380(20):1929–1940. 31091374 10.1056/NEJMoa1813904

[88] Grenn FP, Kim JJ, Makarious MB, The Parkinson’s Disease Genome-Wide Association Study Locus Browser. Mov Disord. 2020;35(11):2056–2067. 32864809 10.1002/mds.28197 PMC7754106

[89] Alvarado CX, Makarious MB, Weller CA, omicSynth: An open multi-omic community resource for identifying druggable targets across neurodegenerative diseases. Am J Hum Genet. 2024;111(1):150–164. 38181731 10.1016/j.ajhg.2023.12.006 PMC10806756

[90] Chang D, International Parkinson’s Disease Genomics Consortium, Nalls MA, A metaanalysis of genome-wide association studies identifies 17 new Parkinson’s disease risk loci. Nat Genet. 2017;49(10):1511–1516. 28892059 10.1038/ng.3955 PMC5812477

[91] Finan C, Gaulton A, Kruger FA, The druggable genome and support for target identification and validation in drug development. Sci Transl Med. 2017;9(383). doi:10.1126/scitranslmed.aag1166 PMC6321762 28356508

