# Supplementary figures and images for: Prioritizing Parkinson’s disease risk genes in genome-wide association loci
Source: medRxiv. 2024 Dec 14:2024.12.13.24318996. Preprint. [Version 1] doi: 10.1101/2024.12.13.24318996 (PMC11661345; doi:10.1101/2024.12.13.24318996)

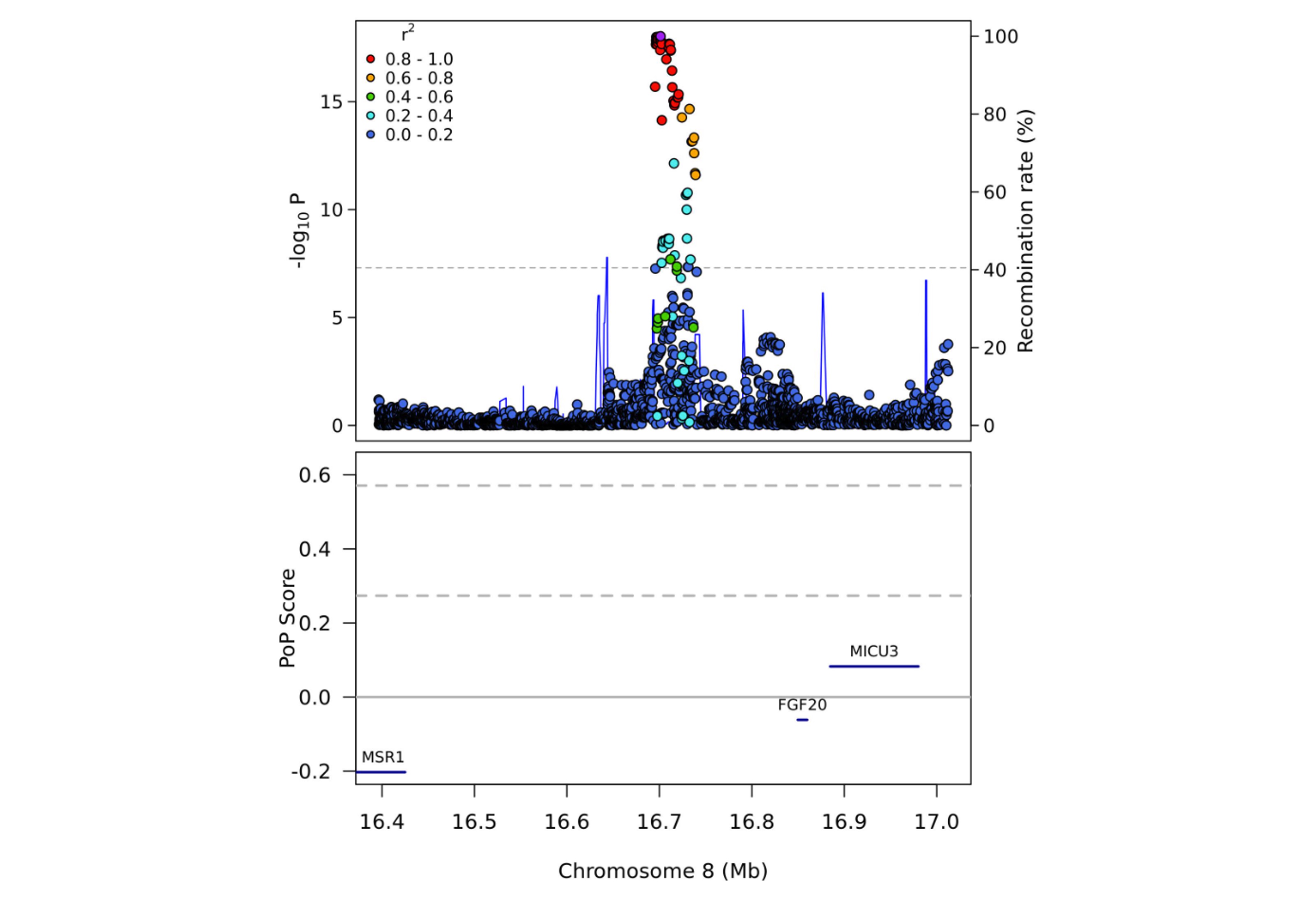

Supplement: Supplement 2 — Figure S1. Variant-level associations and PoPS results for the MICU3 locus. The upper portion of each sub-plot is a LocusZoom plot. Each point represents a different genetic variant, the x-axis represents physical position on the listed chromosome, the left y-axis represents −log10-transformed P value, the right y-axis represents the recombination rate, colour represents linkage disequilibrium with the lead variant in the locus (as shown in the legend), and the horizontal dashed line represents the genome-wide significance P value threshold of 5×10^−8^. The lower portion of each figure is a PoPS plot. Genes are denoted as blue bars spanning from their transcription start site to their transcription stop site using the same x-axis as the LocusZoom plot, the y-axis represents the raw PoPS score, the dashed horizontal grey lines represent the top 10% and 1% of PoPS scores genome-wide, and the solid horizontal grey line represents a PoPS score of 0. [file media-2.jpg]
